# Encorafenib and binimetinib followed by radiotherapy for patients with BRAF^V600^-mutant melanoma and brain metastases (E-BRAIN/GEM1802 phase II study)

**DOI:** 10.1093/neuonc/noae116

**Published:** 2024-07-01

**Authors:** Iván Márquez-Rodas, Ana Álvarez, Ana Arance, Izaskun Valduvieco, Miguel-Ángel Berciano-Guerrero, Raquel Delgado, Ainara Soria, Fernándo Lopez Campos, Pedro Sánchez, Jose Luis Romero, Juan Martin-Liberal, Anna Lucas, Roberto Díaz-Beveridge, Antonio-José Conde-Moreno, Maria del Carmen Álamo de la Gala, Almudena García-Castaño, Pedro José Prada, María González Cao, Enrique Puertas, Joana Vidal, Palmira Foro, Carlos Aguado de la Rosa, Juan Antonio Corona, Pablo Cerezuela-Fuentes, Paco López, Pablo Luna, Neus Aymar, Teresa Puértolas, Pilar Sanagustín, Alfonso Berrocal

**Affiliations:** Department of Medical Oncology, Hospital Universitario Gregorio Marañón, Madrid, Spain; Department of Radiation Oncology, Hospital Universitario Gregorio Marañón, Universidad Complutense, Madrid, Spain; Department of Medical Oncology, Hospital Clínic Barcelona, Barcelona, Spain; Department of Radiation Oncology, Hospital Clínic Barcelona, Barcelona, Spain; Medical Oncology Intercenter Unit, Hospitales Universitarios Regional y Virgen de la Victoria de Málaga, IBIMA-Plataforma BIONAND, Málaga, Spain; Department of Radiation Oncology, Hospital Universitario Regional de Málaga, Málaga, Spain; Department of Medical Oncology, Hospital Universitario Ramón y Cajal, Madrid, Spain; Department of Radiation Oncology, Hospital Universitario Ramón y Cajal, Madrid, Spain; Department of Medical Oncology, Hospital Universitario Reina Sofía, Córdoba, Spain; Department of Radiation Oncology, Hospital Universitario Reina Sofía, Córdoba, Spain; Department of Medical Oncology, Institut Catalá d’Oncologia (ICO) L’Hospitalet de Llobregat, Barcelona, Spain; Department of Radiation Oncology, Institut Catalá d’Oncologia (ICO) L’Hospitalet de Llobregat, Barcelona, Spain; Department of Medical Oncology, Hospital Universitario y Politécnico La Fe de Valencia, Valencia, Spain; Department of Radiation Oncology, Hospital Universitario y Politécnico La Fe de Valencia, Valencia, Spain; Department of Medical Oncology, Hospital Universitario Virgen de la Macarena, Sevilla, Spain; Department of Medical Oncology, Hospital Universitario Marqués de Valdecilla, Santander, Spain; Department of Radiation Oncology, Hospital Universitario Marqués de Valdecilla, Santander, Spain; Department of Medical Oncology, Hospital Universitari Dexeus, Instituto Oncológico Dr. Rosell, Barcelona, Spain; Department of Radiation Oncology, Hospital Universitario QuirónSalud Dexeus,Barcelona, Spain; Department of Medical Oncology, Hospital del Mar, Barcelona, Spain; Department of Radiation Oncology, Hospital del Mar, Barcelona, Spain; Department of Medical Oncology, Hospital Clínico San Carlos, Madrid, Spain; Department of Radiation Oncology, Hospital Clínico San Carlos, Madrid, Spain; Department of Medical Oncology, Hospital Clínico Universitario (HCU) Virgen de la Arrixaca; IMIB. Ciudad de Murcia, Spain; Department of Radiation Oncology, Hospital Clínico Universitario (HCU) Virgen de la Arrixaca; IMIB. Ciudad de Murcia, Spain; Department of Medical Oncology, Hospital Universitario Son Espases, Palma de Mallorca, Spain; Department of Radiation Oncology, Hospital Universitario Son Espases, Palma de Mallorca, Spain; Department of Medical Oncology, Hospital Universitario Miguel Servet, Zaragoza, Spain; Department of Radiation Oncology, Hospital Universitario Miguel Servet, Zaragoza, Spain; Department of Medical Oncology, Hospital General Universitario de Valencia, Valencia, Spain

**Keywords:** brain metastasis, encorafenib and binimetinib, melanoma, radiotherapy, targeted therapy

## Abstract

**Background:**

Encorafenib plus binimetinib (EB) is a standard-of-care treatment for advanced BRAF^V600^-mutant melanoma. We assessed the efficacy and safety of encorafenib plus binimetinib in patients with BRAF^V600^-mutant melanoma and brain metastasis (BM) and explored if radiotherapy improves the duration of response.

**Methods:**

E-BRAIN/GEM1802 was a prospective, multicenter, single-arm, phase II trial that enrolled patients with melanoma BRAF^V600^-mutant and BM. Patients received encorafenib 450 mg once daily plus binimetinib 45 mg BID, and those who achieved a partial response or stable disease at first tumor assessment were offered radiotherapy. Treatment continued until progression. Primary endpoint was intracranial response rate (icRR) after 2 months of EB, establishing a futility threshold of 60%.

**Results:**

The study included 25 patients with no BM symptoms and 23 patients with BM symptoms regardless of using corticosteroids. Among them, 31 patients (64.6%) received sequential radiotherapy. After 2 months, icRR was 70.8% (95% CI: 55.9–83.1); 10.4% complete response. Median intracranial progression-free survival (PFS) and OS were 8.5 (95% CI: 6.4–11.8) and 15.9 (95% CI: 10.7–21.4) months, respectively (8.3 months for icPFS and 13.9 months OS for patients receiving RDT). Most common grades 3–4 treatment-related adverse event was alanine aminotransferase (ALT) increased (10.4%).

**Conclusions:**

Encorafenib plus binimetinib showed promising clinical benefit in terms of icRR, and tolerable safety profile with low frequency of high-grade TRAEs, in patients with BRAF^V600^-mutant melanoma and BM, including those with symptoms and need for steroids. Sequential radiotherapy is feasible but it does not seem to prolong response.

**Key Points**

- E + B with/without RDT have potential benefits in BRAF^V600^-mut melanoma and BM patients.
- ORR of 60.9% in patients with neurological symptoms, suggesting an alternative when ICI is contraindicated.
- Sequential RDT does not seem to prolong response.

**Importance of the Study**

The experience in the treatment of patients with advanced BRAF-mutated melanoma and brain metastasis using encorafenib plus binimetinib remains limited. The E-BRAIN/GEM1802, a prospective, multicenter, single-arm, phase II trial evaluated the intracranial response rate, progression-free survival (PFS), overall survival (OS), and safety outcomes in patients receiving encorafenib plus binimetinib, with focus on the role of radiotherapy in prolonging response. Our findings suggested that the combination therapy is significantly beneficial and tolerable for patients with BRAF V600-mutant melanoma and brain metastasis, with radiotherapy demonstrating safety without an observable increase in toxicity. Our findings provide novel insights into the treatment strategies for this challenging patient population and contribute significantly providing valuable insights into the efficacy of encorafenib plus binimetinib, especially for patients for whom immunotherapy is contraindicated or not effective.

The presence of brain metastases in patients with melanoma is highly prevalent and associated with poor prognosis, including its own dedicated M category subclassification in the AJCC 8th edition.^1^ Targeted therapy (for patients with *BRAF*^*V600*^-mutant melanoma) and immunotherapy have demonstrated activity in patients with melanoma and brain metastases. Based on the results of the ABC^2^ and checkmate204^3^ trials, Ipilimumab and nivolumab are the preferred option especially for patients with asymptomatic brain metastases, showing an intracranial response rate (icRR) of up to 57% and prolonged overall survival (OS) that compares similarly to patients without brain metastases in pivotal clinical trials.^4^ However, patients with symptomatic brain metastases and/or the need for steroids exhibited poorer outcomes, with an icRR of 16.7% and a median progression-free survival (PFS) of less than 2 months.^3^ For patients with *BRAF*^*V600*^-mutant melanoma and brain metastases, the combination of dabrafenib and trametinib in the COMBI-MB clinical trial showed a high icRR (up to 59%), regardless of the presence of symptoms.^5^ However, the duration of response, PFS and OS were shorter than those observed with ipilimumab and nivolumab in asymptomatic patients and with dabrafenib and trametinib in patients without brain metastases.^6^ Other combinations, such as vemurafenib and cobimetinib with atezolizumab, have been recently explored in patients with *BRAF*^*V600*^-mutant melanoma and brain metastases (including those with symptoms), with an icRR of 42% (35% for symptomatic patients) and a median PFS of 5.5 months.^7^

According to the COLUMBUS clinical trial, the combination of encorafenib and binimetinib has demonstrated superiority in both PFS and OS in patients with advanced *BRAF*^*V600*^-mutant melanoma in comparison with vemurafenib.^8^ However, patients with brain metastases untreated locally and stable were excluded. To date, only few retrospective studies, most of which included patients who previously underwent targeted therapy, have investigated the intracranial activity of encorafenib and binimetinib.^9^ Thus, prospective evaluation of the intracranial activity of encorafenib and binimetinib in targeted therapy naïve patients with brain metastases remains unknown.

In the COMBI-MB clinical trial, the cohort B including patients with previously locally treated brain metastases (including radiotherapy) exhibited better 1-year PFS compared to the other cohorts (47% vs. 8%–19%, respectively), leading to a plausible hypothesis that local treatment could improve the duration of response,^5^ which is critical for targeted therapy in comparison with immunotherapy.

Accordingly, we designed the current phase II clinical trial to prospectively assess the intracranial activity of encorafenib and binimetinib in patients with *BRAF*^*V600*^-mutant melanoma and brain metastases (with or without symptoms). In addition, the study has the exploratory objective to evaluate whether performing brain radiotherapy after 2 months of induction treatment with encorafenib and binimetinib could improve the duration of response.

## Patients and Methods

### Study Population and Procedures

The EBRAIN (GEM-1802) was a prospective, multicenter, single-arm, phase II trial led by the Spanish Multidisciplinary Melanoma Group (GEM; clinicaltrials.gov NCT03898908). The study was conducted at 15 sites in Spain.

Supplementary Figure 1 summarizes the study design, including the treatment and efficacy evaluation plans. The study included adult (≥18 years old) patients with *BRAF*^*V600*^-mutant melanoma detected locally by validated tests, brain metastases, an Eastern Cooperative Oncology Group (ECOG) of ≤ 2, at least one RECIST-modified measurable lesion (5 mm in the longest diameter by magnetic resonance imaging (MRI) but no lesions larger than 50 mm), and no prior targeted therapy for advanced melanoma. Previously targeted therapies were permitted only in the adjuvant setting. Previous immunotherapy was allowed in both adjuvant and advanced settings, before developing brain metastasis (BM). Previous local surgery was allowed but previous brain radiotherapy (RDT) was not allowed. Antiepileptic and supportive care treatments were allowed in cases where no major interactions with encorafenib and binimetinib were present. Corticosteroids for control of symptoms were allowed, with no dose limit. Patients with only leptomeningeal involvement were excluded.

Initially, 2 cohorts of patients with *BRAF*^*V600*^-mutant melanoma and brain metastases were planned: One main cohort of patients with asymptomatic brain metastases at enrollment and one exploratory cohort of patients with symptomatic brain metastases. Due to slow accrual and the results from previous studies in patients with MBM^5^ and a non-preplanned interim analysis^10^ showing that no differences in icRR between the 2 cohorts were expected, the steering committee decided to analyze both asymptomatic and symptomatic as one single cohort. Moreover, this decision was undertaken due to the evolving and changing definition of symptomatic that may limit the interpretation of the results as already discussed in recent studies.^7^

The patients were treated with standard doses of encorafenib (450 mg PO every 24 hours) and binimetinib (45 mg PO every 12 hours) for 2 months, after which a brain MRI and body computed tomography (CT) scan were performed. If according to the investigator, no signs of progressive disease were detected in either the brain or body, brain RDT was offered to the patients, with the exceptions of: Intracranial complete response (CR), in which RDT was spared for further progressive disease; or patient’s refusal to be treated with RDT (in this later case after discussing with medical monitor). RDT treatment was recommended but not compulsory. The different RDT techniques, fractions, and doses were decided as per institutional guidelines according to the investigator’s team radiation oncologist and a guidance summarized in Supplementary Table 1. RDT schemes different from those proposed in current guidelines were discussed with the radiation oncology coordinator.

Encorafenib and binimetinib were discontinued 24 hours before, during and 24 hours after RDT, and then continued until disease progression, unacceptable toxicity, death, or the patient’s decision. Brain MRI and body CT scan were performed every 2 months during the first year, and as per institutional guidelines thereafter. Demographic patient characteristics and previous cancer history were documented at baseline. Patients underwent clinical and physical examinations including documentation of vital signs, ECOG performance status, and laboratory assessments (including hematologic blood counts, blood chemistry panel, and urinalysis) every 4 weeks during treatment and at the safety visit (within 28 days after the last dose of study treatment). The blood chemistry panel included: lactate dehydrogenase (LDH), CK (CPK), gamma-glutamyltransferase (GGT), creatinine, creatinine clearance, total bilirubin, alkaline phosphatase, aspartate aminotransferase (AST) or GOT, alanine aminotransferase (ALT) or GPT, calcium (Ca), potassium (K), and magnesium (Mg). EORTC QLQ 30 quality of life (QoL) questionnaires were completed before any study-specific determination at the following visits: baseline, week 8 (after the first 2 months of treatment with encorafenib and binimetinib), and week 24. Basic neurological assessments according to clinical practice and the Modified Barthel Index were collected at baseline, and weeks 4, 8, 24, and every 4 weeks thereafter until disease progression. Safety was assessed by means of frequency and severity of adverse events (AEs) according to the Common Terminology Criteria for AEs (CTCAE—version 4.03) at each patient visit (continuously) from the time informed consent was signed until the end of the study. The causality and outcomes for each AE were assessed and reported by the principal investigator. All AEs were followed until resolution. Cardiac function was assessed through standard 12-lead ECG at baseline, after 4 weeks of treatment, after every 12 weeks, and at the safety visit. Additional ECGs may be performed at the discretion of the investigator, and LFEV was assessed at baseline and as per institutional guidelines. Concomitant medications were recorded continuously from 30 days before starting the trial until the end of the study. All women with reproductive capacity must have a negative urine pregnancy test within 72 hours before receiving study treatment and the pregnancy tests were repeated every 4 weeks until the end of treatment.

### Objectives

The initial primary endpoint was to assess the efficacy of the study treatment by means of icRR at 2 months after starting treatment with encorafenib and binimetinib (prior to any dose of radiotherapy), as evaluated by the investigator and according to the modified RECIST criteria (mRECIST, see Supplementary Table 2) in asymptomatic patients. No confirmation of responses was requested, as depending on the first tumor assessment patients could undergo RDT and this would have interfered with tumor assessments. After a non-preplanned analysis, the protocol was amended to evaluate as the primary objective the icRR according to mRECIST with asymptomatic and symptomatic patients together.

Secondary objectives included assessing the efficacy by means of intracranial PFS (icPFS), extracranial PFS (ecPFS), OS, QoL, and safety in the global cohort and disaggregated into 2 post hoc subgroups: (1) patients with asymptomatic brain metastases and no need for steroids for symptoms control; and (2) patients that at the time of treatment initiation had neurological symptoms either uncontrolled or controlled with corticosteroids. icPFS was defined as the time from inclusion (first dose of treatment) until intracranial tumor progression (mRECIST). ecPFS was defined as the time from inclusion (first dose of treatment) until extracranial tumor progression (RECIST 1.1) or death. Patients without any of the previous events are censored at the date of the last available tumor assessment.

### Statistical Methods

The required sample size was initially calculated for the primary endpoint, the intracranial response rate, for the cohort of asymptomatic patients using Fleming’s single-stage procedure. Investigators assessed icRR in COMBI-MB, a benchmark study for TKIs in patients with melanoma BM, varied across subgroups from 44 to 59%.^5^ Therefore, we hypothesized that the experimental treatment would achieve an icORR *≤*40% (null hypothesis), and an icORR of ≥60% was adopted as an alternative hypothesis. Employing an alpha error of 0.05 and a power of 80%, the expected total sample size required was 38 patients. Factoring in an estimated 20% attrition due to loss of follow-up before day 56 without undergoing the first tumor assessment, the initial sample size was set at 48 patients, to ensure obtaining 38 evaluable patients. The cohort of symptomatic patients was exploratory and no formal sample calculation was performed, anticipating the inclusion of up to 15 patients. An interim analysis revealed no differences between cohorts.^10^ Consequently, the trial was amended and the icORR was analyzed irrespective of symptomatology in all enrolled patients. The null and alternative hypothesis for sample size calculations were maintained for the pooled analysis of all patients based on evidence from previous studies and our own interim report showing no differences in ORR between symptomatic and asymptomatic patients.^5,10^ Thus, the trial required a total of 38 evaluable patients to maintain adequate statistical power to observe differences as initially proposed.

The efficacy analysis was based on the intention-to-treat population, including all patients enrolled in the trial. Additional analysis was performed in the per-protocol population, including all patients fulfilling all eligibility criteria and having at least 2 valid tumor assessments (baseline and one evaluation post-baseline by MRI or CT-scan) without any protocol deviation, invalidating the patient for primary endpoint evaluation. A secondary analysis was also performed in 2 groups defined post hoc based on their symptomatology and the use of corticosteroids. Comparisons between patients who received RDT or not were post hoc. Safety was assessed on all patients who received at least one dose of treatment. Descriptive statistics were used to evaluate baseline demographic characteristics. Continuous variables were summarized using descriptive statistics (n, median, mean, standard deviation, range, or 95% confidence interval [CI]), as applicable. Categorical data were represented as frequency counts and percentages of patients within each category. Time-to-event endpoints (icPFS, ecPFS, and OS) were estimated using the Kaplan–Meier method.

All statistical analyses were performed using R and SPSS softwares (IBM SPSS Statistics Version 26). Figures and tables were generated using RStudio (Version 1.2.5033 2009-2019 RStudio).

### Ethical Considerations

The E-BRAIN / GEM-1802 study was approved by independent ethics committees and competent authorities in Spain (Agencia Española del Medicamento y Productos Sanitarios, AEMPS), and performed in accordance with the Declaration of Helsinki, International Conference on Harmonization Guidelines for Good Clinical Practice, and applicable local laws. Written informed consent was obtained from all patients before to study enrollment. This study was registered in EudraCT (2018-002530-20) and www.clinicaltrials.gov (NCT03898908).

### Data Availability

The Supplementary Information file contains the study protocol. Additional data are available from the corresponding author upon reasonable request. Data will be provided anonymously, with no identifiable data.

## Results

### Patient Characteristics

Between July 2019 and October 2022, 48 patients were enrolled, with 47 (97.9%) patients receiving a minimum of 2 months of treatment and being evaluable for the primary endpoint (Supplementary Figure 2). One patient discontinued the study treatment after the first month following the investigator criteria. The study analyzed descriptively also 2 post hoc subgroups: (1) totally asymptomatic patients [*N* = 25], and (2) patients with neurological symptoms before start of treatment, either non-controlled or controlled with corticosteroids [*N* = 23] Patient characteristics are described in Table 1. Among these 23 symptomatic patients, corticosteroids were used prior to study entry for symptom control in 8 (34.8%) patients achieving symptom disappearance and 14 (61%) patients with persistent symptomatology. Among asymptomatic patients, 9 (36%) received corticosteroids prior to study entry for reasons unrelated to intracranial symptoms, primarily for the management of previous toxicities from immunotherapy in 5 patients (20%), asymptomatic perilesional edema in 2 (8%), arthritis in 1 (4%), and symptomatic spinal metastasis in 1 (4%). A detailed description of the symptoms present in patients is provided in Supplementary Table 3.

**Table 1.** Patient Characteristics

Characteristic		Total *N* = 48
median Age (range); years		54 (18–88)
Sex; *n* (%)	Male	24 (50)
	Female	24 (50)
ECOG PS; *n* (%)	0	26 (54.2)
	1	20 (41.7)
	2	2 (4.2)
Barthel index; *n* (%)	Total dependent (0–4)	3 (6.3)
	Severe dependent (5–12)	4 (8.3)
	Moderate dependent (13–18)	6 (12.5)
	Slight dependent (19–20)	33 (68.9)
	NA	2 (4.2)
BRAF genotype; *n* (%)*	V600E	41 (87.2)
	V600K	11 (22.9)
	V600R	6 (12.5)
	V600 other	25 (52.1)
Brain symptoms; *n* (%)	Asymptomatic	25 (52.1)
	Symptomatic	23 (47.9)
Median SLD of target intracranial lesions (range); mm		26.5 (6–134)
Num of brain target lesion; *n* (%)	1	21 (43.8)
	2	15 (31.3)
	3 or more	12 (25)
Mean num of brain target lesions (range)		2 (1–5)
Extracranial metastases; *n* (%)	Yes	41 (85.4)
	No	7 (14.6)
LDH, *n* (%)	≤ULN	26 (54.2)
	>ULN	21 (43.8)
	Unknown	1 (2.1)
Baseline corticosteroids before first dose of study treatment *n* (%)	Yes	33 (68.8)
	No	15 (31.2)
Median steroids dose before first dose of study treatment (dexametasone equivalent) mg (range) per day		8 (1–16)
Previous anti-PD-1 based immunotherapy; *n* (%)	Anti PD-1	11 (22.9)
	Anti PD-1 / anti CTLA-4	5 (10.4)
	No	32 (66.7)
Radiotherapy received in the EBRAIN trial; *n* (%)	RS	10 (20.8)
	FSRT	6 (12.5)
	WBRT	15 (31.2)
	No	17 (35.4)

Abbreviations: ECOG PS, Eastern Cooperative Oncology Group Performance Status; RS, radiosurgery; FSRT, fractionated stereotactic radiotherapy; LDH, lactate dehydrogenase; SLD, sum of largest diameters; ULN, upper limit normal; WBRT, whole brain radiotherapy. # Had no symptoms related to brain metastasis and received corticosteroids for other reasons. * Patients may harbor more than one mutation and/or the analytical technique detects the presence of one out of several V600 alterations.

### Primary Endpoint: Intracranial Response Rate According to mRECIST

**Figure 1.**
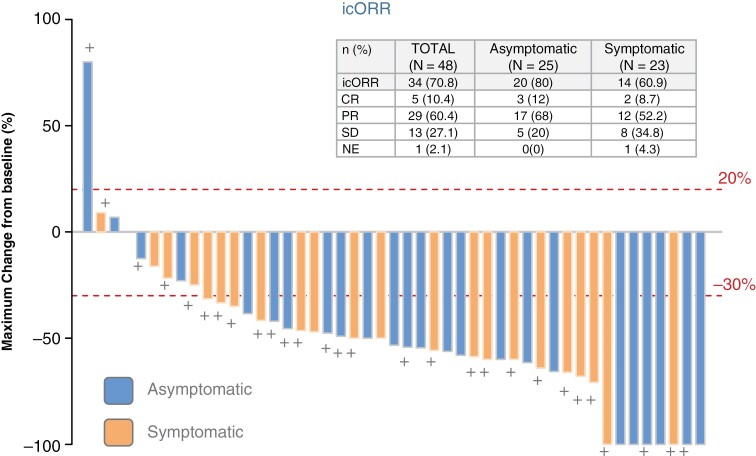
Confirmed maximum reduction in intracranial target lesion (at any time) in patients with symptomatic and asymptomatic brain metastasis. Dashed red lines at 20% and –30% represent the threshold of progression and partial response respectively. Crosses mark patients who were receiving corticosteroids at baseline. 1 patient was not evaluated for response.

With a median follow-up (reverse censoring) of 24.9 months (95% CI: 21.8), the icRR at 2 months (before radiotherapy, in the first assessment) was 70.8% (95% CI: 55.9–83.1), with 5 (10.4%) CR, and 29 (60.4%) partial responses (PR). Patients with stable disease (SD) were 13 (27.1%) and 1 (2%) patient was not evaluated because he withdrew from the study before the assessment (Figure 1). Response rate was 80% in neurologically asymptomatic patients and 60.9% in symptomatic patients. The median duration of response was 5.6 (95% CI: 3.6–7.5) months.

### Secondary Endpoints

**Figure 2.**
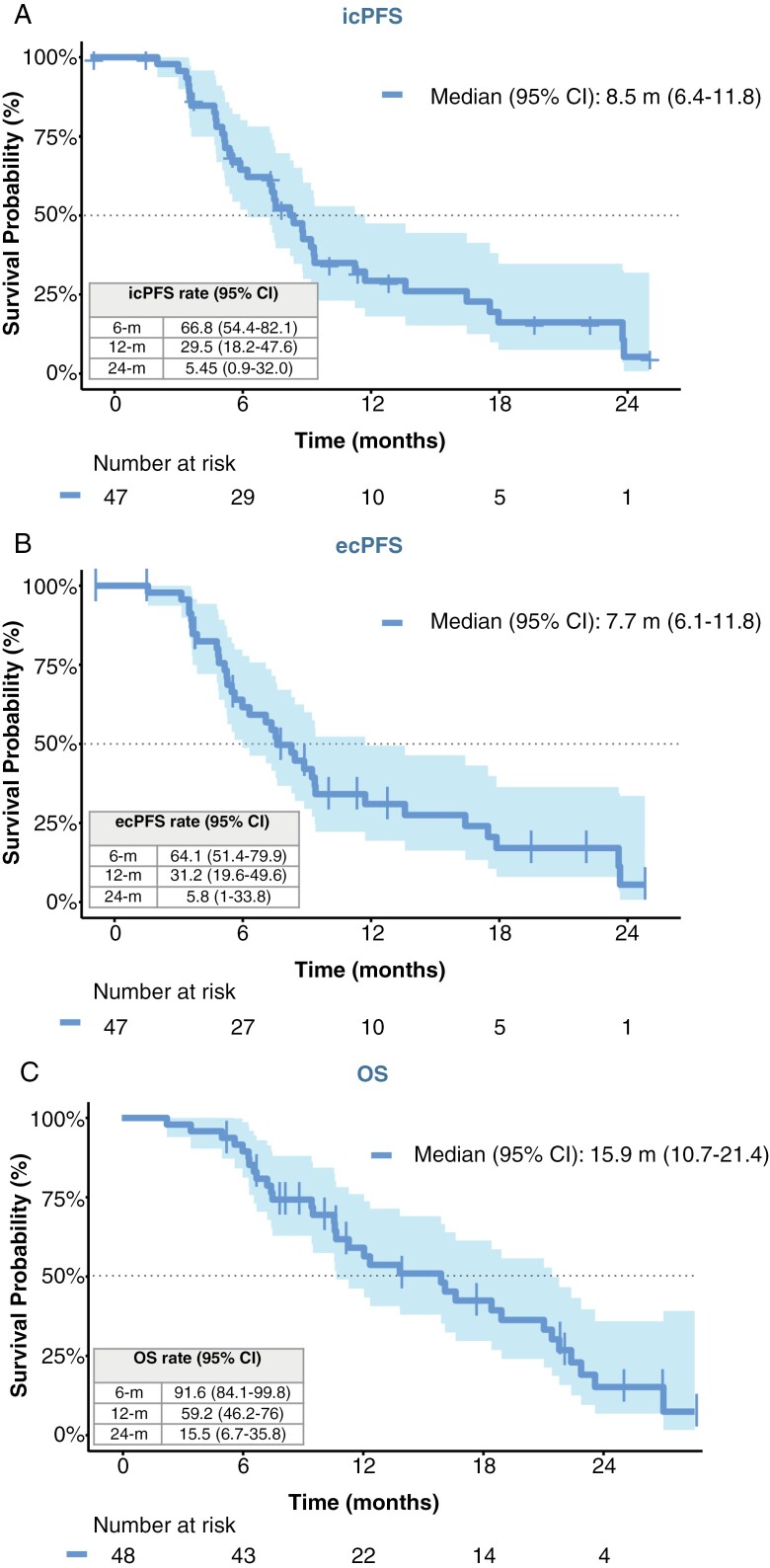
Kaplan–Meier graphs showing intracranial PFS (A), extracranial PFS (B), and OS (C) in the overall population. Median survivals for each subgroup are included in the graph. Censored patients are marked with a cross line. Dashed lines represent the 50% rate of events.

The median icPFS was 8.5 (95% CI: 6.4–11.8) months with 29.5% free of progression at one year (Figure 2A). Regarding extracranial disease, median ecPFS was 7.7 (95% CI: 6.1–11.8) months (Figure 2B). Median OS was 15.9 (95% CI: 10.7–21.4) and 59.2% of patients were alive at 1 year (Figure 2C). Four patients remained alive 2 years after the start of the study treatment. Supplementary Figure 3 illustrates the results of icPFS, ecPFS, and OS in the 2 post hoc subgroups.

RDT was offered to patients achieving partial response or SD, aiming to prolong those responses. A total of 31 (64.6%) patients underwent radiotherapy, with 16 (33.3%) receiving localized therapies and 15 (31.3%) undergoing whole brain radiotherapy (WBRT). There were 11 patients who were eligible for RDT according to protocol recommendations but did not start the local treatment following the investigator’s decision. The median icPFS was 8.3 (95% CI: 6.1–9.5) months and 16.4 (95% CI: 5.4–NR) months for patients receiving and not receiving RDT, respectively (Supplementary Figure 4A). The patients who received RDT showed a duration of response of 5.6 months (95% CI: 3–7.5). The median OS was 13.9 (95% CI:10.6–21) months and 22.4 (95% CI: 10.6–NR) months for patients receiving and not receiving RDT, respectively (Supplementary Figure 4B). The 1-year icPFS rate was 20.8% and 54.1%; the 1-year OS rate was 56.7% versus 64.8% for patients receiving and not receiving RDT, respectively.

Encorafenib and binimetinib were discontinued in 44 (91.7%) patients at the time of the analysis (November 2023), mainly due to: disease progression 31 (64.6%), toxicities 6 (12.5%), investigator decision 3 (6.3%), patient consent withdrawal 2 (4.2%), and surgical intervention 2 (4.2%). Treatment-related AEs leading to discontinuation of the study treatment included transaminitis, muscle weakness, anemia, creatinine increase, general discomfort, and cardiac toxicity (decrease in LVEF that coursed with muscle weakness and edema in lower limbs). Temporary treatment interruptions and dose reductions for AEs management were reported in 24 (50%) and 13 (27.1%) patients, respectively. The AEs that led to dose modification were mainly transaminitis (10.4%), and diarrhea (6.3%).

**Figure 3.**
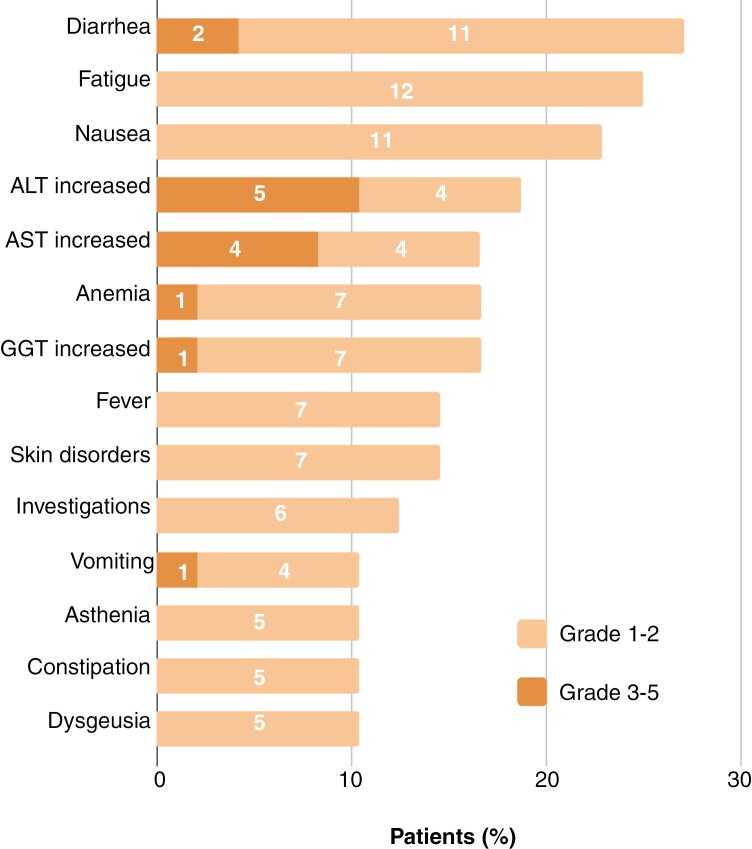
Most common treatment-related adverse events. Bars represent the percentage of patients who experience each event type. Numbers within the bar show the number of patients who had each event. The minimum frequency threshold used for the figure is 10%.

The most frequent treatment-related AEs were diarrhea (27%), fatigue (25%), nausea (22.9%), elevated alanine aminotransferase (ALT), and aspartate aminotransferase (AST; 18.8% and 16.7%, respectively). Most events were low-grade and were appropriately managed with dose modifications or rescue medications. Grade 3–4 toxicities were reported in 12 (25%) patients, all related to encorafenib and binimetinib and none to radiotherapy. The most common grades 3–4 were elevated ALT (10.4%), elevated AST (8.3%), and diarrhea (4.2%). No toxic deaths were reported. Figure 3 and Supplementary Table 4 summarize treatment-related AEs (encorafenib and binimetinib, RDT, or both) according to the investigator´s criteria.

Baseline QoL questionnaires were completed by 35 (72.9%) patients. The overall QLQ-C30 score exhibited a significant transient improvement at week 8 (*P* = .015) after treatment initiation (Supplementary Figure 5). The global health status showed no statistically significant improvement at weeks 8 and 24. Notably, in patients with symptoms (*N* = 15), the global health status showed an improvement that was statistically significant at weeks 8 (*P* = .046) and 24 (*P* = .022; Supplementary Figure 5). Consistent with this result, the QLQ-C30 score showed a significant transient improvement at week 8 (*P* = .033), while physical, social, and role functioning showed no statistically significant improvement. A significant improvement in insomnia was reported at week 8 (*P* = .004) and was maintained at week 24 (*P* = .02) in symptomatic patients. Conversely, no changes in insomnia were reported in asymptomatic patients, who already had lower insomnia levels than baseline that were maintained. Symptoms such as pain, fatigue, and appetite loss showed non-significant progressive improvement from baseline in the follow-up visits. Changes observed in QoL showed a similar trend in patients who underwent radiotherapy of any type, or WBRT specifically. No significant worsening was observed with WBRT (Supplementary Figure 6).

## Discussion

This is the final analysis of the EBRAIN / GEM-1802 trial, which constitutes to our knowledge the first prospective study that shows intracranial activity from encorafenib and binimetinib, in addition to the feasibility of combination with radiotherapy. The primary endpoint of intracranial response rate was achieved, with 70.8% of both asymptomatic and symptomatic patients exhibiting a response to treatment and surpassing the expected futility threshold. Intracranial response rate was in line with previous reports using BRAF and MEK inhibitors in patients with metastatic melanoma with BM.^5^ Despite the good response rates, the duration of the response was shorter than in patients without BM, 5.6 months versus 18.6 months reported in the COLUMBUS trial, this is also true for OS (15.9 months in our study versus 33.6 months respectively).^8^ These differences in PFS and OS were also present when comparing dabrafenib and trametinib in patients with^5^ and without^6^ brain metastases in pivotal clinical trials (COMBI-MB).

Intracranial responses were observed in patients with neurological symptoms and also in patients with concomitant use of corticosteroids, suggesting that the use of corticosteroids likely did not interfere with the efficacy observed in this trial. This is particularly noteworthy as it remains a limitation with immunotherapies, especially in symptomatic patients.^11^ The observed intracranial response rate was higher than that reported for immune checkpoint inhibitors.^2,3^ Moreover, triple combinations of immune checkpoint inhibitors with BRAF/MEK inhibitors (atezolizumab plus vemurafenib plus cobimetinib) showed comparable or lower icRR^7^ than those in the current study. However, the prognosis of patients with symptoms and need for corticosteroid therapy is still poor and will require the implementation of novel therapeutic approaches.

The safety profile of encorafenib and binimetinib in this study was similar to that reported in previous studies in patients with metastatic melanoma.^8^ Gastrointestinal events secondary to treatment were predominantly mild, with only 2 patients suffering grade 3 diarrhea, effectively managed with dose modifications/interruptions. Pyrexia occurred in 14.6% of patients, a lower rate than observed with other BRAF / MEK inhibitor combinations such as dabrafenib and trametinib.^5^ Thus, the combination of encorafenib and binimetinib demonstrated a manageable safety profile in patients with melanoma BM. Patient self-reported outcomes, also strengthened the safety profile, and exhibited significant improvement in symptoms and global status in patients with symptoms, who had worse self-perceived health status at baseline.

Regarding the additional value of radiotherapy for patients with intracranial PR or SD, our findings indicate the feasibility of this combination in terms of safety. No new safety concerns were raised with the combination. However, given the exploratory nature of this objective, and the absence of clear differences between patients who received brain radiotherapy and those who did not, which may be the consequence of a potential selection bias, no conclusions for the recommendation for this radiation approach are feasible.

The main limitation of this study lies in its non-randomized design, precluding direct comparisons with immunotherapy and other BRAF / MEK inhibitors currently approved for the treatment of patients with metastatic melanoma. The performance of an interim analysis may have an impact on type I errors. The small sample size of subgroups only allowed an exploratory analysis for hypothesis generation. Moreover, these subgroup analyses were performed post hoc and were not statistically powered to observe differences so we could not discard effects which are not seen. The lack of standardization in radiotherapy techniques introduced variability between sites. In addition, some patients were given WBRT even though current guidelines suggest that its use should be restricted to carefully selected patients.^12^ Nevertheless, no concerns were raised from QoL in patients who underwent WBRT. Another limitation, common with other clinical trials dedicated to patients with melanoma and brain metastases, is the efficacy criteria used. In our case, we decided to use the modified RECIST criteria used in the COMBI-MB clinical trial, since this clinical trial also treated patients with BRAF and MEK inhibitors (dabrafenib and trametinib). Whether other evaluation criteria dedicated to brain involvement such as Response Assessment in Neuro-Oncology for BM (RANO-MB)^13^ could better evaluate the outcomes of patients with melanoma and brain metastases is a question to explore in future clinical trials. Central imaging review would have reinforced our findings.

Lastly, we concluded that in contrast with immunotherapy, the response to encorafenib and binimetinib was independent of the presence of symptoms. Although the data collection design and investigator assessment aimed to unequivocally indicate the clear presence or absence of symptoms related to brain metastases, the definition, and categorization of these symptoms lack a globally recognized international consensus. This was another study limitation that led to a change in study design to combine the initial 2 cohorts to avoid the classification of patients according to symptoms. Therefore, future studies involving patients with brain metastases, especially those experiencing symptoms, would benefit from the establishment of a standardized and internationally accepted framework for the harmonization of symptom definitions.

In conclusion, our findings provide evidence that encorafenib plus binimetinib have a potential clinically significant benefit in patients with *BRAF*^*V600*^-mutant melanoma and BM, especially in patients with symptomatic MBM, and maintain tolerable safety profiles. These findings serve as a framework for future research and confirm the usefulness of BRAF and MEK inhibitors in brain metastases as an alternative treatment, particularly in cases where immunotherapy failed, is contraindicated, or is expected to be less effective.

## Supplementary material

Supplementary material is available online at *Neuro-Oncology* (https://academic.oup.com/neuro-oncology).

noae116_suppl_Supplementary_Material

## Prior Presentation

Presented as oral communication at the ESMO Annual Meeting 2021 and a poster at 2 meetings: the ESMO Annual Meeting 2022 and the ESTRO Annual Meeting 2023.

## References

[1] Gershenwald JE , Scolyer RA, Hess KR, et al; for members of the American Joint Committee on Cancer Melanoma Expert Panel and the International Melanoma Database and Discovery Platform. Melanoma staging: Evidence-based changes in the American joint committee on cancer eighth edition cancer staging manual: Melanoma staging: AJCC 8 ^th^ Edition. CA Cancer J Clin. 2017;67(6):472–492. 29028110 10.3322/caac.21409 PMC5978683

[2] Long GV , Atkinson V, Lo S, et al. Five-year overall survival from the anti-PD1 brain collaboration (ABC Study): Randomized phase 2 study of nivolumab (nivo) or nivo+ipilimumab (ipi) in patients (pts) with melanoma brain metastases (mets). J Clin Oncol. 2021;39(15_suppl):9508–9508.

[3] Tawbi HA , Forsyth PA, Hodi FS, et al. Long-term outcomes of patients with active melanoma brain metastases treated with combination nivolumab plus ipilimumab (CheckMate 204): Final results of an open-label, multicentre, phase 2 study. Lancet Oncol. 2021;22(12):1692–1704. 34774225 10.1016/S1470-2045(21)00545-3 PMC9328029

[4] Wolchok JD , Chiarion-Sileni V, Gonzalez R, et al. Overall survival with combined nivolumab and ipilimumab in advanced melanoma. N Engl J Med. 2017;377(14):1345–1356. 28889792 10.1056/NEJMoa1709684 PMC5706778

[5] Davies MA , Saiag P, Robert C, et al. Dabrafenib plus trametinib in patients with BRAFV600-mutant melanoma brain metastases (COMBI-MB): A multicentre, multicohort, open-label, phase 2 trial. Lancet Oncol. 2017;18(7):863–873. 28592387 10.1016/S1470-2045(17)30429-1 PMC5991615

[6] Robert C , Grob JJ, Stroyakovskiy D, et al. Five-year outcomes with dabrafenib plus trametinib in metastatic melanoma. N Engl J Med. 2019;381(7):626–636. 31166680 10.1056/NEJMoa1904059

[7] Dummer R , Queirolo P, Gerard Duhard P, et al. Atezolizumab, vemurafenib, and cobimetinib in patients with melanoma with CNS metastases (TRICOTEL): A multicentre, open-label, single-arm, phase 2 study. Lancet Oncol. 2023;24(12):e461–e471. 37459873 10.1016/S1470-2045(23)00334-0

[8] Dummer R , Flaherty KT, Robert C, et al. COLUMBUS 5-Year Update: A randomized, open-label, phase III trial of encorafenib plus binimetinib versus vemurafenib or encorafenib in patients with BRAF V600-mutant melanoma. J Clin Oncol. 2022;40(36):4178–4188. 35862871 10.1200/JCO.21.02659 PMC9916040

[9] Holbrook K , Lutzky J, Davies MA, et al. Intracranial antitumor activity with encorafenib plus binimetinib in patients with melanoma brain metastases: A case series. Cancer. 2020;126(3):523–530. 31658370 10.1002/cncr.32547 PMC7004095

[10] Marquez-Rodas I , Arance A, Guerrero MAB, et al. 1038MO Intracranial activity of encorafenib and binimetinib followed by radiotherapy in patients with BRAF mutated melanoma and brain metastasis: Preliminary results of the GEM1802/EBRAIN-MEL phase II clinical trial. Ann Oncol. 2021;32(5):S870.

[11] Tawbi HAH , Forsyth PAJ, Hodi FS, et al. Efficacy and safety of the combination of nivolumab (NIVO) plus ipilimumab (IPI) in patients with symptomatic melanoma brain metastases (CheckMate 204). J Clin Oncol. 2019;37(15_suppl):9501–9501.

[12] Keilholz U , Ascierto PA, Dummer R, et al. ESMO consensus conference recommendations on the management of metastatic melanoma: Under the auspices of the ESMO guidelines committee. Ann Oncol. 2020;31(11):1435–1448. 32763453 10.1016/j.annonc.2020.07.004

[13] Lin NU , Lee EQ, Aoyama H, et al; Response Assessment in Neuro-Oncology (RANO) group. Response assessment criteria for brain metastases: Proposal from the RANO group. Lancet Oncol. 2015;16(6):e270–e278. 26065612 10.1016/S1470-2045(15)70057-4

