# Supplementary material for: Encorafenib and binimetinib followed by radiotherapy for patients with BRAFV600-mutant melanoma and brain metastases (E-BRAIN/GEM1802 phase II study)
Source: Neuro Oncol. 2024 Jul 1;26(11):2074–83. doi: 10.1093/neuonc/noae116 (PMC11534317; doi:10.1093/neuonc/noae116)
Supplement: noae116_suppl_Supplementary_Material [file noae116_suppl_supplementary_material.docx]

**SUPPLEMENTARY INFORMATION**

**Supplementary figure 1. Study Scheme.** All patients received COMBO450 (encorafenib 350 mg QD and binimetinib 45 mg BID) for at least 56 days of treatment, and were evaluated with a contrast enhanced brain MRI and CT body scan at first tumor assessment. Patients with PD at first evaluation in brain MRI (per RECIST modified criteria) and /or body CT scan (per RECIST 1.1 criteria) discontinued study drugs and were followed for overall survival. Patients with intracranial complete response (CR) and no systemic PD, were followed every two months with brain MRI and body CT scan up to 12 months, continued treatment with COMBO450 and local treatment could be administered to patients with an intracranial CR following the physician criteria. Patients with no CR nor PD in brain MRI per modified RECIST 1.1 criteria, and no systemic PD per RECIST 1.1, were treated with local radiotherapy (RT) (radiosurgery or WBRT), and then continued with the COMBO 450.

**
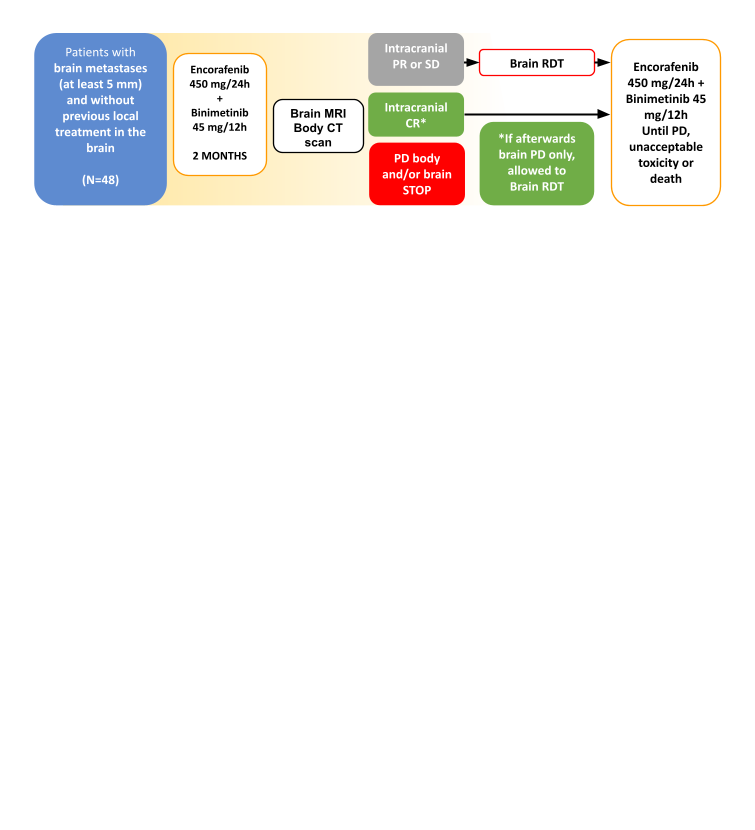
**

**Supplementary figure 2. Consort diagram showing the patient distribution according to their symptoms.** * Corticosteroid administered for other reasons than control of symptoms related to melanoma brain metastasis. Abbreviations: CR, complete response; FAS, full analysis set; PI, principal investigator; RS, radiosurgery; SP, safety population; SRS, stereotactic radiosurgery; WBRT, whole brain radiotherapy.


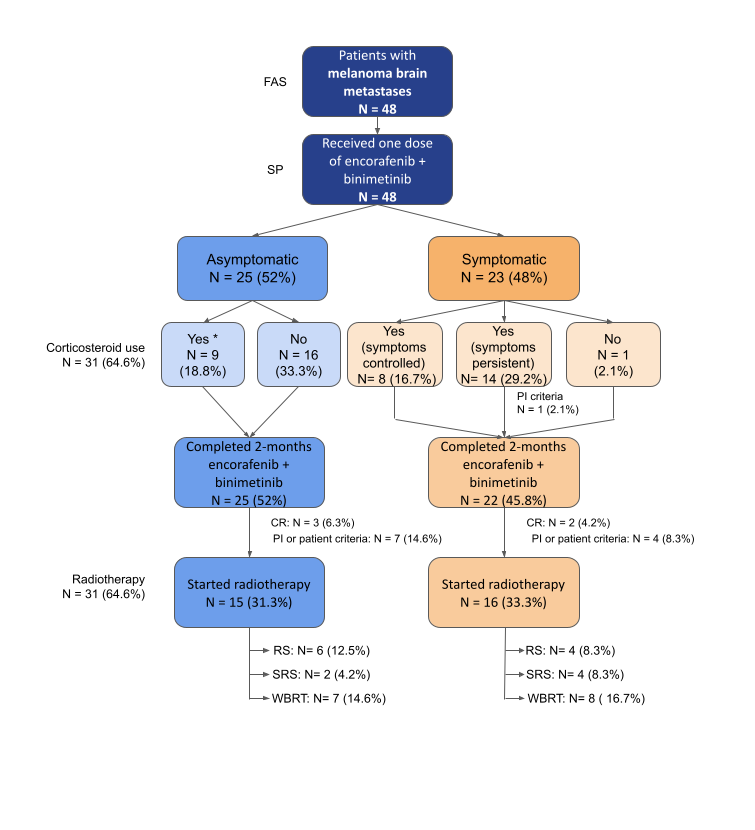


**Supplementary figure 3. Kaplan Meier graphs showing intracranial PFS (A), extracranial PFS (B) and overall survival (C) in patients stratified in the *post hoc* groups according to their symptomatology.** Median survivals for each subgroup are included in the graph. This analysis was performed *post hoc*. Censored patients are marked with a cross line. Dashed lines represent the 50% rate of events.


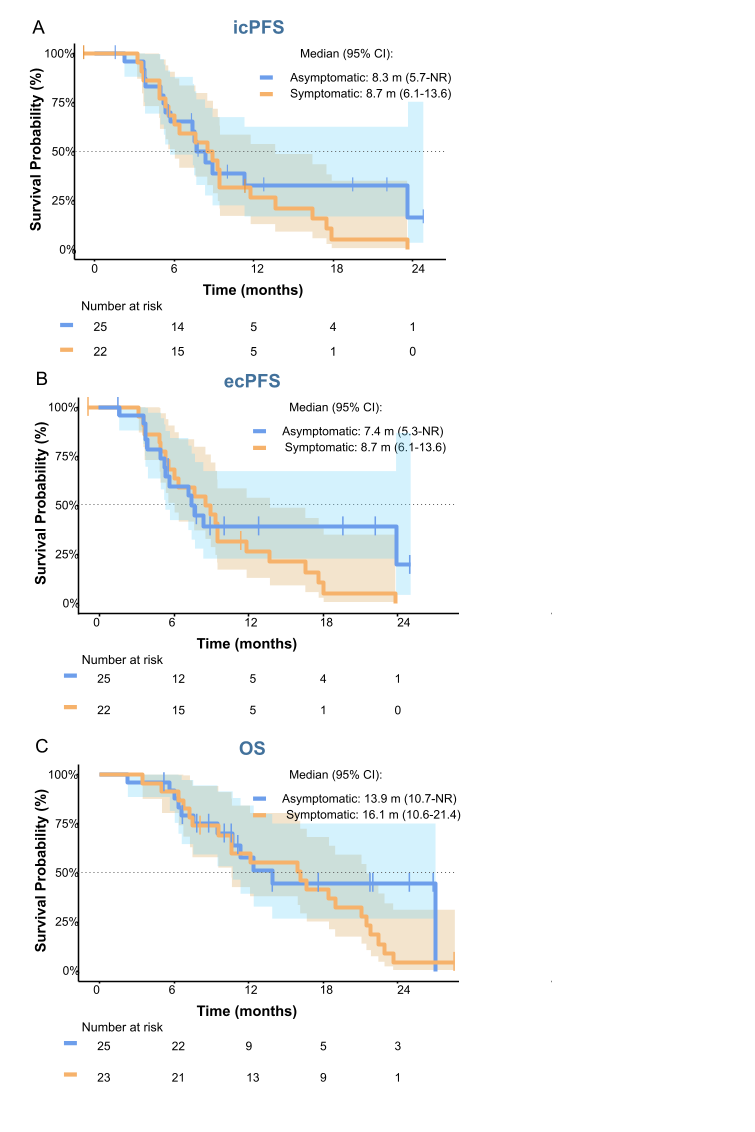


**Supplementary figure 4. Kaplan Meier graphs showing intracranial PFS (A) and OS (B) in patients stratified according to the administration of radiotherapy.** Median survivals for each subgroup are included in the graph. This analysis was performed *post hoc*. Censored patients are marked with a cross line. Dashed lines represent the 50% rate of events.


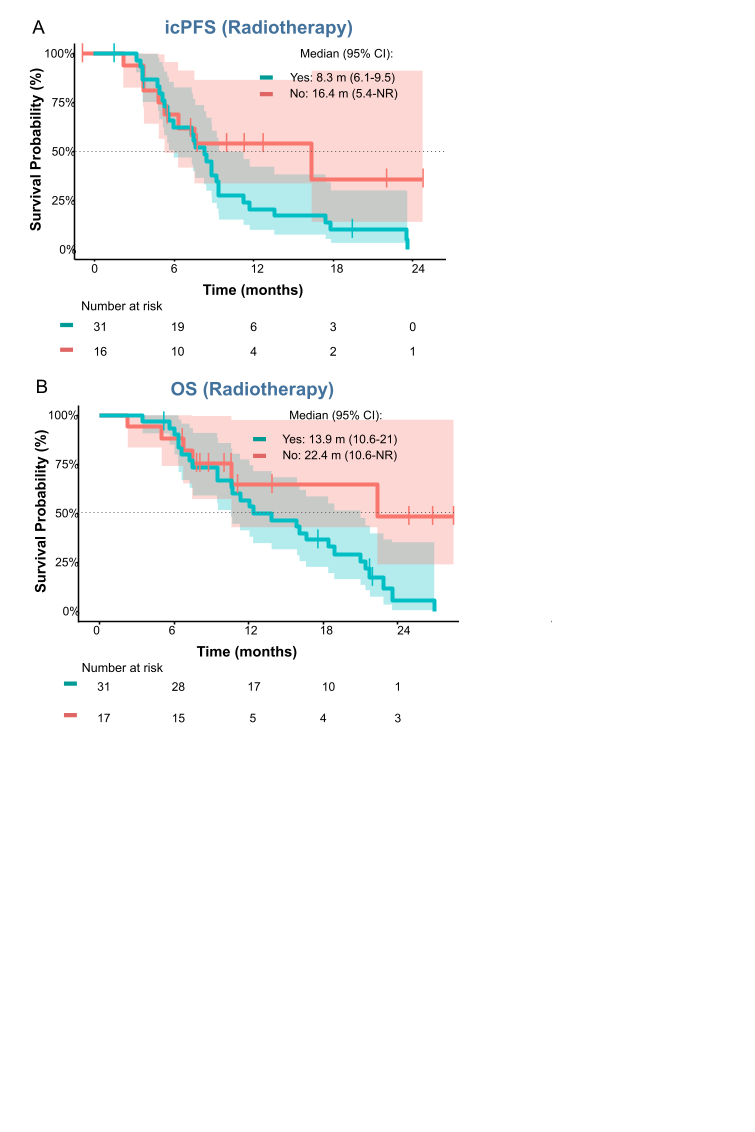


**Supplementary figure 5. Quality of life.** Global QLQ-C30 score (A) and statistical significant formulaires are depicted. B) Global health status; C) Insomnia. Patients with symptomatic brain metastasis (Orange) and asymptomatic (Blue). The QLQ-C30 questionnaire was obtained before the first dose of study treatment (baseline), after the first stage of two months of encorafenib-binimetinib treatment (week 8) and after radiotherapy if performed or 2 additional months of therapy (week 24). Boxplots show median and interquartile range, 95% CI is depicted with the interval and outliers represented as dots. Scale ranges from 0 to 100, higher values indicate better performance for status (A and B) and worse for symptoms (C). Statistically significant differences from baseline are indicated with a * (*P<0.05*), with the cohort color if differences were present in a specific cohort or black if present also for the full dataset.


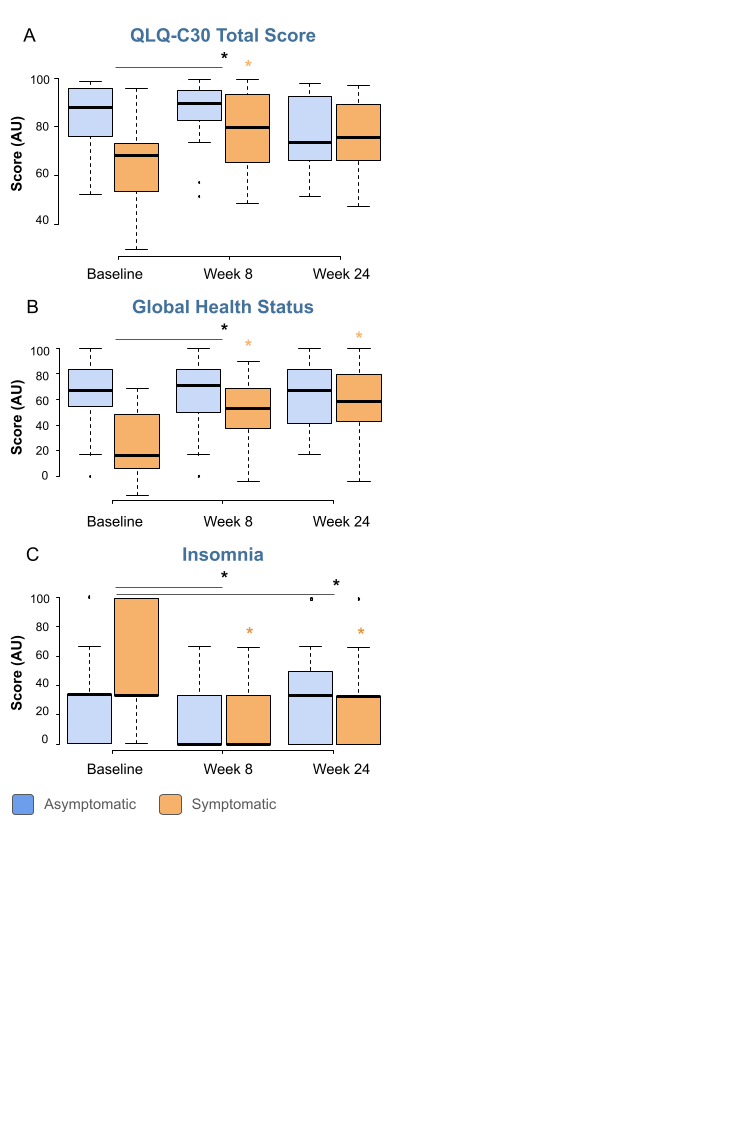


**Supplementary figure 6. Quality of life in patients receiving radiotherapy.** Global QLQ-C30 score (A) and statistical significant formulaires are depicted. B) Global health status; C) Insomnia. Patients with symptomatic brain metastasis (Orange) and asymptomatic (Blue). The QLQ-C30 questionnaire was obtained before the first dose of study treatment (baseline), after the first stage of two months of encorafenib-binimetinib treatment (week 8) and after radiotherapy if performed or 2 additional months of therapy (week 24). Boxplots show median and interquartile range, 95% CI is depicted with the interval and outliers represented as dots. Scale ranges from 0 to 100, higher values indicate better performance for status (A and B) and worse for symptoms (C). Statistically significant differences from baseline are indicated with a * (*P<0.05*), with the color of the subpopulation if differences were present in a specific patient subset.

**
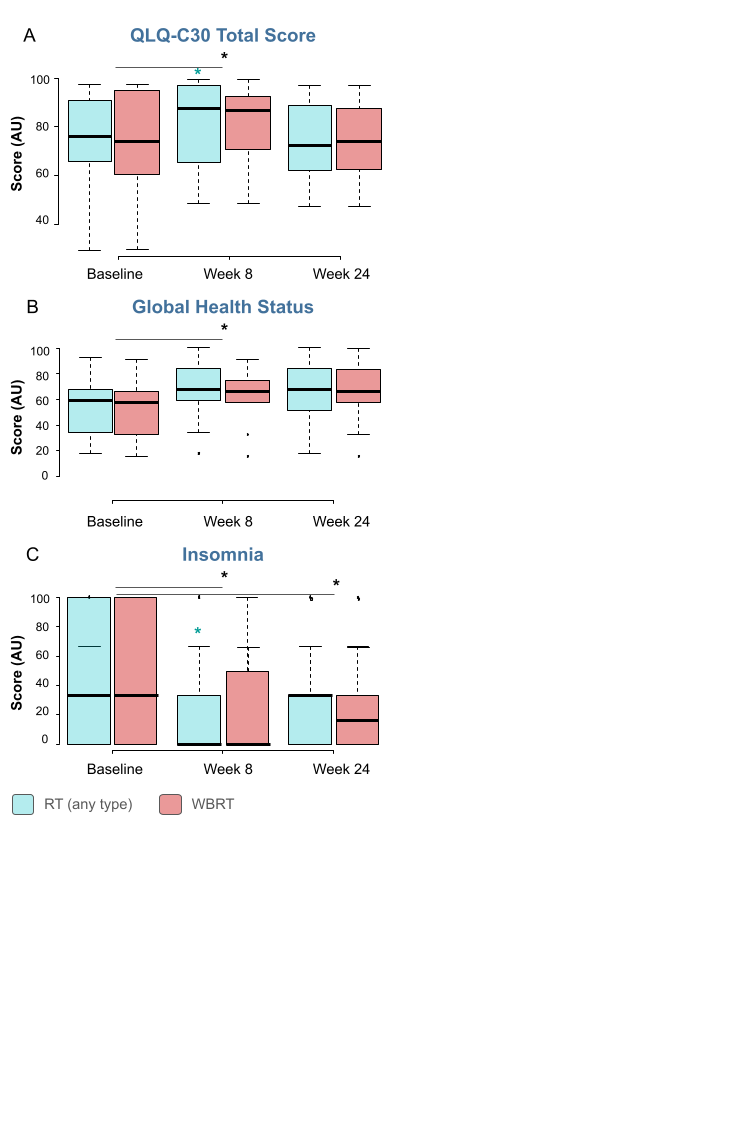
**

**Supplementary Table 1. Guidance for RDT techniques.**

| **Patient profile** | **Recommended radiotherapy technique** |
| --- | --- |
| - - - - 1 brain metastases < 4 cm       - ≤ 3 brain metastases ≤ 3 cm       - ≤ 4 brain metastases ≤ 2.5 cm | Radiosurgery (RS) or  Stereotactic radiosurgery (SRS) |
| - - - - ≥ 4 brain metastases       - Almost one brain metastasis of ≥ 4 cm       - ≤ 4 brain metastases not eligible for RS or SRS       - Metastases located at brain stem, midbrain, pons, and medulla or within 10 mm of the optic nerves and chiasm. | Whole brain radiotherapy (WBRT) |

**Supplementary Table 2. Modified RECIST criteria.** The main changes to the standard RECIST version 1.1 criteria are listed.

| **Criteria** | | **Changes from RECIST 1.1** |
| --- | --- | --- |
| Number of target lesions | Intracranial | - Up to 5 intracranial lesions should be selected as target; based on size and evaluation/tracking feasibility. All the remaining brain lesions different from these 5 target lesions must be considered as non-target lesions (independently of their size). The size of target and non-target lesions must be measured. |
|  | Extracranial | - The number of lesions was in accordance with RECIST 1.1 |
| Measurability definitions * | Intracranial | - Measurable implies that it can be accurately measured at least in one dimension with the longest diameter ≥ 5 mm when evaluated by contrast-enhanced MRI. - Non-measurable intracranial lesion: all other lesions, including those too small to be considered measurable (longest diameter < 5 mm). - Measurable intracranial disease: The presence of at least one measurable lesion. - Non-measurable intracranial (only) disease: The presence of only non-measurable lesions, which at baseline excludes the patient for participation in the study. |
|  | Extracranial | - Measurability criteria for lesions was in accordance with RECIST 1.1 |

* After local treatment, intracranial lesions (both target and non-target lesions) will be considered as non-evaluable (NE).

**Supplementary Table 3. Patient symptomatology.** A patient may have more than one symptom at baseline.

| **Symptom; n (%)** | **EBRAIN-MEL**  **N = 48** | **Asymptomatic #**  **N = 25** | **Symptomatic**  **N = 23*** |
| --- | --- | --- | --- |
| Headache | 9 (18.8) | 0 (0) | 9 (39.1) |
| Epilepsy | 3 (6.3) | 0 (0) | 3 (13) |
| Motor deficit | 7 (14.6) | 0 (0) | 7 (30.4) |
| Sensory deficit | 2 (4.2) | 0 (0) | 2 (8.7) |
| Speech déficit | 3 (6.3) | 0 (0) | 3 (13) |
| Vomits | 2 (4.2) | 0 (0) | 2 (8.7) |
| Fever | 1 (2.1) | 0 (0) | 1 (4.3) |

* Symptoms were controlled with a stable or decreasing dose of corticosteroids.

* 22 patients received corticosteroids for the management of their symptomatology.

# Had no symptoms related to brain metastasis. 9 patients received corticosteroids for control of toxicities from previous treatments.

**Supplementary Table 4. Encorafenib plus binimetinib toxicity profile.** A cutoff minimum frequency threshold of 5% was used.

| **Adverse event; n (%)** | **G 1-2** | **G 3-5** | **Overall** |
| --- | --- | --- | --- |
| Diarrhea | 11 (22.9) | 2 (4.2) | 13 (27.1) |
| Fatigue | 12 (25) | 0 (0) | 12 (25) |
| Nausea | 11 (22.9) | 0 (0) | 11 (22.9) |
| ALT increased | 4 (8.3) | 5 (10.4) | 9 (18.8) |
| AST increased | 4 (8.3) | 4 (8.3) | 8 (16.7) |
| Anemia | 7 (14.6) | 1 (2.1) | 8 (16.7) |
| GGT increased | 7 (14.6) | 1 (2.1) | 8 (16.7) |
| Pyrexia | 7 (14.6) | 0 (0) | 7 (14.6) |
| Skin and subcutaneous tissue disorders | 7 (14.6) | 0 (0) | 7 (14.6) |
| Investigations | 6 (12.5) | 0 (0) | 6 (12.5) |
| Vomiting | 4 (8.3) | 1 (2.1) | 5 (10.4) |
| Asthenia | 5 (10.4) | 0 (0) | 5 (10.4) |
| Constipation | 5 (10.4) | 0 (0) | 5 (10.4) |
| Dysgeusia | 5 (10.4) | 0 (0) | 5 (10.4) |
| Gastrointestinal disorders | 4 (8.3) | 0 (0) | 4 (8.3) |
| Arthralgia | 4 (8.3) | 0 (0) | 4 (8.3) |
| Musculoskeletal and connective tissue disorder | 4 (8.3) | 0 (0) | 4 (8.3) |
| CPK increased | 2 (4.2) | 1 (2.1) | 3 (6.3) |
| Eye disorders | 3 (6.3) | 0 (0) | 3 (6.3) |
| Pruritus | 3 (6.3) | 0 (0) | 3 (6.3) |
| Abdominal pain | 3 (6.3) | 0 (0) | 3 (6.3) |
| Creatinine increased | 3 (6.3) | 0 (0) | 3 (6.3) |
| Lymphocyte count decreased | 3 (6.3) | 0 (0) | 3 (6.3) |
